# Noncoding RNAs in the Interplay between Tumor Cells and Cancer-Associated Fibroblasts: Signals to Catch and Targets to Hit

**DOI:** 10.3390/cancers13040709

**Published:** 2021-02-09

**Authors:** Martina Tassinari, Paolo Gandellini

**Affiliations:** Department of Biosciences, University of Milan, 20133 Milan, Italy; martina.tassinari@unimi.it

**Keywords:** cancer-associated fibroblast, extracellular vesicles, long noncoding RNA, liquid biopsy, microRNA, therapeutic target, therapy resistance

## Abstract

**Simple Summary:**

Cancer aggressiveness is the result of a proficient bidirectional interaction between tumor and stromal cells within the tumor microenvironment, among which a major role is played by the so-called cancer-associated fibroblasts. Upon such interplay, both cancer cells and fibroblasts are reprogrammed to sustain malignancy, with changes in the repertoire of noncoding RNAs, mainly microRNAs and long noncoding RNAs. Such molecules are also exchanged between the two cell types through extracellular vesicles. In this review, we summarize the current knowledge of microRNAs and long noncoding RNAs that act intracellularly or extracellularly to sustain tumor-stroma interplay. We also provide our view regarding the possible clinical utility of such noncoding RNAs as therapeutic target/tools or biomarkers to predict patient outcome or response to specific treatments.

**Abstract:**

Cancer development and progression are not solely cell-autonomous and genetically driven processes. Dynamic interaction of cancer cells with the surrounding microenvironment, intended as the chemical/physical conditions as well as the mixture of non-neoplastic cells of the tumor niche, drive epigenetic changes that are pivotal for the acquisition of malignant traits. Cancer-associated fibroblasts (CAF), namely fibroblasts that, corrupted by cancer cells, acquire a myofibroblast-like reactive phenotype, are able to sustain tumor features by the secretion of soluble paracrine signals and the delivery extracellular vesicles. In such diabolic liaison, a major role has been ascribed to noncoding RNAs. Defined as RNAs that are functional though not being translated into proteins, noncoding RNAs predominantly act as regulators of gene expression at both the transcriptional and post-transcriptional levels. In this review, we summarize the current knowledge of microRNAs and long noncoding RNAs that act intracellularly in either CAFs or cancer cells to sustain tumor-stroma interplay. We also report on the major role of extracellular noncoding RNAs that are bidirectionally transferred between either cell type. Upon presenting a comprehensive view of the existing literature, we provide our critical opinion regarding the possible clinical utility of tumor-stroma related noncoding RNAs as therapeutic target/tools or prognostic/predictive biomarkers.

## 1. Introduction

Cancer development and progression are not dependent solely on the genetic make-up of tumor cells neither are cell-autonomous mechanisms. Dynamic interaction of cancer cells with the surrounding microenvironment, intended as the chemical/physical conditions (hypoxia, mechanical stress, composition of the extracellular matrix) as well as the complex mixture of non-neoplastic cells (fibroblasts, endothelial cells, normal epithelial cells, pericytes, mesenchymal stem cells and immune cells) of the tumor niche, drive epigenetic changes that are pivotal for the acquisition of malignant traits [1].

**Figure 1:**
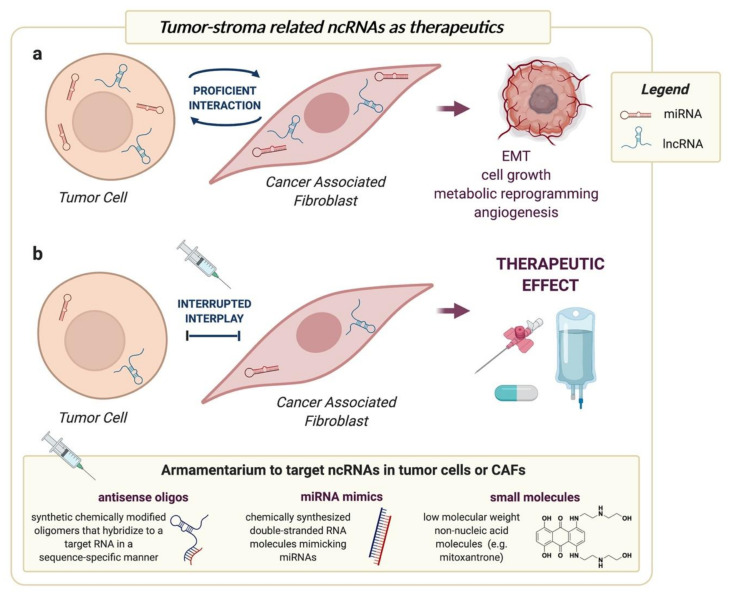
ncRNAs shown to play a role in either fibroblast activation or in CAF-induced changes in recipient cancer cells may be regarded as possible therapeutic targets or tools to interrupt tumor-stroma interplay. (**a**) The proficient interaction between tumor cells and CAFs sustains malignant features, such as EMT, cell growth, metabolic reprogramming and angiogenesis, through changes in the repertoire of both miRNAs and lncRNAs; (**b**) Strategies to overexpress or inhibit ncRNAs, including antisense oligonucleotides, miRNA mimics or small molecules, can be used to interrupt tumor-stroma interplay to obtain therapeutic benefits in terms of direct anticancer effect or enhanced response to conventional treatments. Created with BioRender.com.

One of the most abundant cell types within tumor microenvironment is represented by cancer-associated fibroblasts (CAF), namely the fibroblasts that, corrupted by cancer cells through the secretion of cytokines and growth factors (e.g., TGF-β, IL-6), acquire a myofibroblast-like phenotype resembling that of wound healing reactive stroma [2]. In a diabolic liaison, CAFs are in turn able to induce malignant features in tumor cells by the secretion of soluble paracrine signals and the delivery extracellular vesicles [3,4]. CAF-induced changes cover almost all of the hallmarks of cancer, such as the increased cell proliferation, epithelial-mesenchymal transition (EMT) with enhanced invasion capabilities, angiogenesis and metabolic reprogramming (Figure 1a). In addition, CAFs can also dramatically remodel the extracellular matrix, thus ultimately favor cancer cell dissemination and metastasis [3].

In the bidirectional interplay between tumor cells and CAFs, a major role has been ascribed to noncoding RNAs (ncRNAs). Defined as RNAs that are functional though not being translated into proteins, ncRNAs predominantly act as regulators of gene expression at both the transcriptional and post-transcriptional levels [5]. Different classes of ncRNAs have been described, depending on the size, structure and mode of action (reviewed in [5]). In the cancer field, the last 20 years have witnessed a huge amount of discoveries regarding the key role of microRNAs (miRNAs) [6]. They are small 22–23 nt long RNAs that in normal cells are appointed to finely tune protein output through a post-transcriptional gene silencing mechanism. In fact, by binding to at least partially complementary sequences in target mRNAs (mainly within 3’-UTR), they can induce mRNA degradation or inhibit translation [7]. Plenty of evidence has been collected to show that the aberrant miRNA expression found in almost all types of human cancer is not only the consequence rather one of the causes of transformation and tumor progression [8,9,10]. In more recent years, defects in the functioning of other ncRNAs have also been associated with cancer. Among these, relevant for this paper are long noncoding RNAs (lncRNAs) [11]. LncRNAs comprise all ncRNAs longer than 200 nt. Though some of them share features typical of mRNAs, such as 5’ capping, 3’ polyadenylation and alternative splicing, lncRNAs have null or very limited protein-coding potential [12]. So far, only a few lncRNAs have been ascribed with specific biological functions, thus giving rise to an intense debate on whether the majority of them may be just non-functional byproducts of transcription [13]. In the current definition, functional lncRNAs are a very heterogeneous class of nuclear or cytoplasmic RNAs able to regulate gene expression at multiple levels—transcriptional, post-transcriptional, and post-translational—, in different directions—positively or negatively—, and using disparate modes of action. Exploiting their nucleic acid nature together with the ability to fold into complex secondary and tertiary structures, lncRNAs can promiscuously interact with DNA, RNA and proteins, thus becoming able to act as scaffolds, decoys, guides or signals [12]. Investigation in the field of lncRNAs is quickly expanding and examples of their involvement in cancer are continuously increasing [14].

Overall, the observed altered function of different classes of ncRNAs in human tumors highlight how cancer is a “disease of dysregulation”. In this scenario, it is not surprising that changes arising in both tumor cells and CAFs upon mutual interaction are driven at the transcriptional and post-transcriptional level by ncRNAs. In this review, we summarize the current knowledge of ncRNAs, mainly miRNAs and lncRNAs, that act intracellularly in either cell type and sustain tumor-stroma interplay. We also report on the major role of extracellular ncRNAs that are bidirectionally transferred between cancer cells and CAFs. Upon presenting a comprehensive view of the existing literature, we provide our critical opinion regarding the possible clinical utility of tumor-stroma related ncRNAs as therapeutic target/tools or prognostic/predictive biomarkers.

## 2. Intracellular ncRNAs Involved in Tumor Cell/CAF Interplay

### 2.1. ncRNAs Modulated in CAFs upon Interaction with Tumor cells

Upon contact with tumor cells, quiescent fibroblasts acquire an activated state which in turn allows them to induce protumorigenic features in cancer cells (Figure 1a). Such acquisition is the result of a dramatic transcriptional and epigenetic reprogramming occurring in fibroblasts, which also involves changes of the noncoding RNA repertoire [2]. In this regard, a seminal report was provided by Mitra and colleagues, who profiled the miRNome (i.e., repertoire of miRNAs) of primary human CAFs isolated from the omental metastases of patients with serous ovarian cancer and compared it with that of fibroblasts extracted from a normal area of the omentum, at least 1 inch from the tumor, from the same patients [15]. In parallel, the authors also profiled miRNAs in normal fibroblasts activated in vitro through co-culture with ovarian cancer cells. The results showed that *miR-31* and *miR-214* were downregulated, whereas *miR-155* was upregulated in both patient-derived and induced CAFs as compared to normal fibroblasts [15]. A similar approach was followed by Doldi and colleagues for prostate cancer CAFs [16]. The authors performed an integrated analysis of miRNA and gene expression in (i) CAFs obtained from tumor tissues of patients subjected to radical prostatectomy, (ii) normal fibroblasts obtained from adjacent non-neoplastic areas, and (iii) the latter activated in vitro with TGF-β or IL-6, two known mediators of fibroblast activation [2]. The miRNAs showing consistent upregulation across all types of activated fibroblasts resulted to be *miR-210*, *miR-143/145* and *miR-590-5p* [16]. Comparative gene expression profiling unveiled similarities between *miR-210* and *miR-590-5p*, thus identifying shared regulated pathways, including the control of translation and insulin receptor recycling. On the other hand, genes correlated with *miR-143* were mainly associated with extracellular matrix and oxidative phosphorylation, in line with the phenotype of activated fibroblasts [16].

The question may then arise as to whether such miRNA modulations are just the downstream effects of other functionally relevant transcriptome changes or if they have a direct role in fibroblast activation. In this regard, Mitra showed that inhibiting *miR-31*/*miR-214* and overexpressing *miR-155* in normal ovarian fibroblasts (thus mimicking the miRNA expression pattern found in CAFs) induced their conversion to a CAF-like state. Notably, the opposite experiment reverted CAFs to normal-like fibroblasts [15]. In line with this, the miRNA-reprogrammed fibroblasts and patient-derived CAFs shared a large number of upregulated genes, mainly chemokines, among which the most expressed was *CCL5* (C-C motif ligand 5), a direct target of *miR-214* [15]. Altogether these results represented to the proof of concept that miRNAs play a direct role in fibroblast activation, so much that fibroblasts may be even reprogrammed through miRNA modulation.

Another miRNA found to be upregulated in both patient-derived and in vitro activated fibroblasts is *miR-210* [16]. Curiously, this miRNA is a direct HIF-1α target and is upmodulated by hypoxia in both tumor cells [17] and senescent fibroblasts [18]. Ectopic overexpression of *miR-210* in young prostate fibroblasts was reported to increase their senescence-associated features and convert them into CAF-like cells, which in turn became able to promote EMT of cancer cells, facilitate the recruitment of monocytes and M2-macrophage polarization, as well as stimulate angiogenesis by mobilizing endothelial precursor cells and enhancing their vasculogenic capability [18].

Similarly to Doldi et al. [16], Melling exposed primary human normal oral fibroblasts to TGF-β1, which resulted in the acquisition of a myofibroblastic CAF-like phenotype. This change was associated with *miR-145* upregulation, a finding that was also confirmed in CAFs derived from oral cancer patients [19]. Apparently in contrast with this, ectopic overexpression of *miR-145* blocked TGF-β1-induced myofibroblastic differentiation and reverted CAF towards a normal fibroblast phenotype, leading the authors to hypothesize that *miR-145* upmodulation is a sort of negative feedback control mechanism against excessive fibroblast activation. In this regard, it is of note that other authors showed that *miR-145* deficiency in the mesenchymal compartment of the intestine (where it is selectively expressed as compared to the epithelial one) leads to dysfunction of smooth muscle and myofibroblast cells, thus suggesting instead a role for *miR-145* in supporting rather than dampening myofibroblastic traits [19].

A miRNA for which a high consensus across studies may be driven is *miR-21*, which was reported as upregulated in CAFs from different tumor histotypes, including pancreatic ductal adenocarcinoma [20,21], lung [22], gastric [23] and breast cancers [24]. In addition, several reports show that *miR-21* is a direct mediator of TGF-β-induced fibroblast activation, as it upregulates several known CAF markers, such as periostin, α-smooth muscle actin, and podoplanin, and stimulates secretion of MMP-3, MMP-9, PDGF, and CCL-7 [21,22]. *miR-21* was also shown to participate to the metabolic reprogramming of CAFs [20], which consists in an enhanced glucose uptake capacity and lactic acid production. Lactate secreted by CAFs is then taken up by tumor cells, which become dependent on it for energy production and cell growth [25]. Inhibition of *miR-21* in CAFs was shown to reduce glycolysis and inhibit their capability to reprogram the metabolism of adjacent tumor cells [20], thus ultimately reducing cancer aggressiveness. Interestingly, in the in vivo setting, overexpression of *miR-21* in CAFs was also shown to promote pancreatic ductal adenocarcinoma desmoplasia and increase drug resistance to gemcitabine treatment, whereas *miR-21* inhibition showed an opposite therapeutic effect [21].

The potential relevance of miRNAs in fibroblast activation is also supported by the evidence that Dicer, the RNAse responsible for processing of the precursor miRNAs, is itself able, when ectopically overexpressed, to induce an inflammatory signature and convert normal ovarian fibroblasts to CAF-like cells able to support malignant features of cancer cells [26].

The involvement of lncRNAs in pathways leading to fibroblast activation has so far been less investigated. In 2017, Vafaee et al. identified 39 unique lncRNAs as differentially expressed in ovarian CAFs with respect to normal fibroblasts, including *MALAT1*, *NEAT1*, *H19*, *GAS5* and *MEG3* [27]. By interrogating known transcription factor-lncRNA interactions and building a context-specific interaction network, they predicted several of these lncRNAs to potentially play a role in metastasis [27].

Other examples of lncRNAs playing a role in CAFs come from the oral squamous cell carcinoma context, where *TIRY* [28] and *FLJ22447* [29] were described as upregulated in CAFs with respect to paracancer fibroblasts. The former was found to act as molecular sponge for *miR-14*, which thus resulted to be depleted in CAF-exosomes and ultimately led to increased invasion and metastasis of oral squamous cell carcinoma cells. The latter, renamed by the authors as *Lnc-CAF*, was shown to upregulate IL-33 levels by preventing its p62-dependent autophagy-lysosome degradation, in turn stimulating tumor cell proliferation.

A comprehensive list of intracellular ncRNAs found to be deregulated in CAFs and/or associated with fibroblast activation is reported in Table 1 (left).

### 2.2. ncRNAs Modulated in Tumor Cells upon Interaction with CAFs

Reprogramming of the ncRNA repertoire is not only responsible for fibroblast activation but also of the phenotypic changes arising in tumor cells upon contact with CAFs. Gandellini et al. [34] profiled miRNAs in prostate cancer cells stimulated with the conditioned media from patient-derived or in vitro induced CAFs and highlighted a major downmodulation of miRNAs known to be repressors of EMT, such as *miR-205* [34] and *miR-200* family members [54]. This result is in line with the evidence that in prostate cancer, the major phenotypic switch induced by CAFs on tumor cells is indeed EMT rather than the enhancement of cell growth [55]. CAF-mediated downregulation of *miR-205* in prostate cancer cells, which resulted to be dependent of a HIF1α-regulated oxidative and proinflammatory cascade [34,56], may also have profound clinical implications for the development of therapy resistance, in light of the fact that the miRNA has been proven to mediate sensitivity of tumor cells both to chemo- and radiotherapy [57,58]. Curiously, unpublished results from our lab show that CAFs are able to induce downregulation of *miR-205* and of the lncRNA derived from *miR-205* host gene *MIR205HG/LEADR* [59] also in normal prostate cells. This may implicate that CAFs that have been activated by tumor cells in a given area of the prostate gland may induce, in a sort of ‘field effect’, pathogenic effects on surrounding normal cells, such as the impairment of basement membrane deposition (regulated by *miR-205*) or an uncontrolled basal-luminal differentiation (regulated by *MIR205HG/LEADR*). CAF-mediated downmodulation of EMT-related miRNAs was also reported for *miR-200b* in gastric cancer cells [33] and *miR-33b* in lung cancer cells [39], and directly associated with the execution of EMT program, enhancement of cell motility and acquisition of stemness features.

Another interesting malignant feature induced by CAFs on cancer cells through the intervention of miRNAs is vascular mimicry, which is the ability to form vascular-like channels instead of endothelial cells, thus providing blood perfusion to the tumor. In this regard, Yang et al. showed that CAFs can promote hepatocellular carcinoma cells to form capillary-like structures in vitro and in vivo through downregulation of *miR-101* [31]. In fact, *miR-101* overexpression was reported to attenuate TGF-β/SDF1 signaling in both CAFs and tumor cells, thus ultimately inhibiting VE-cadherin expression in the latter [31].

LncRNAs have so far been more extensively investigated in the epithelial compartment as compared to the stromal one, with several examples of lncRNAs with altered expression in tumor cells upon CAF interaction. In colorectal cancer, CAFs have been reported to induce upregulation of the human urothelial carcinoma associated 1 (*UCA1*) lncRNA in tumor cells, with subsequent increase of mTOR signaling, suppression of p27 and *miR-143*, and enhanced Cyclin-D1 and KRAS expression. Through this axis, *UCA1* was shown to sustain cancer cell proliferation (evidenced also by an increased percentage of cells in the S and G2/M phase), EMT and migration [47]. In another report, TGF-β1 released by CAFs stimulated the SMAD2/3/4-dependent transcription of *HOTAIR* lncRNA in breast cancer cells. Such lncRNA was found to be implicated in cell proliferation and EMT, as demonstrated by the fact that its depletion inhibited CAF-induced tumor growth and lung metastasis in MDA-MB-231 orthotopic animal model [45]. A similar scenario was evidenced in bladder cancer, where TGF-β1 in CAF-conditioned medium led to phosphorylation of SMAD2 in cancer cells, resulting in the upregulation of *ZEB2NAT*. Such lncRNA demonstrated to be essential for TGFβ1-driven EMT, as *ZEB2NAT* depletion reversed CAF-induced acquisition of mesenchymal features and invasive phenotype, though the reduction of ZEB2 protein levels [50].

Aside from cell proliferation and EMT, metabolic reprogramming in tumors cells is also mediated by the CAF-induced variation of lncRNAs. In this regard, Zhao et al. showed that CXCL14^high^ CAFs determine the upregulation of *LINC00092* in ovarian cancer cells, where the lncRNA increases glycolysis by binding to fructose-2,6-biphosphatase PFKFB2 glycolytic enzyme, thus sustaining the local supportive function of CAFs [46].

A comprehensive list of intracellular ncRNAs found to be deregulated in cancer cells upon interaction with CAFs is reported in Table 1 (right).

## 3. Extracellular ncRNAs Involved in Tumor cell/CAF Interplay

### 3.1. ncRNAs Released by CAFs to Communicate with Tumor Cells

Extracellular vesicles (EVs) are bi-layered membrane vesicles released by almost all cell types into the extracellular space [60]. Their cargo, consisting of specific repertoires of lipids, proteins, mRNAs and ncRNAs, is transferable from donor to recipient cells, where it becomes functionally active. For example, EV-transferred miRNAs are able efficiently downregulate their target mRNAs in receiving cells [61]. Different EV populations may be recognized based on size, density, intracellular origin and biogenesis, including exosomes (30–100 nm diameter), microvesicles (100–1000 nm diameter), large oncosomes (1–10 mm diameter) and apoptotic bodies (1–5 μm diameter) [60]. Exosomes are generated from inward budding of the endosomal membrane; microvesicles, also termed ectosomes, are directly formed from the plasma membrane; large oncosomes are exclusively shed by membrane blebs of cancer cells that acquired an amoeboid phenotype, as an alternative process to induce tumor cell dissemination as compared to the mesenchymal one; apoptotic bodies are released during the disassembly of an apoptotic cell into subcellular fragments [9].

In the context of tumor-stromal interplay, CAF-derived exosomes are the most investigated class of EVs [4]. In general, the exosomal miRNA cargo reflects the miRNA repertoire of the donor cell, although examples of selective sorting of miRNAs into exosomes (i.e., enrichment or depletion of specific miRNAs with respect to the bulk repertoire) have been provided [61]. Exosomes have also been shown to serve as sites for cell-independent miRNA biogenesis, where Dicer can process pre-miRNAs into mature molecules [62].

*miR-21*, showing increased intracellular levels in CAFs compared to normal fibroblasts, was also reported as enriched in exosomes from breast and colon cancer CAFs and directly released to recipient tumor cells [63,64]. Notably, direct transfection of *miR-21* into tumor cells or exposure of tumor cells to exosomes derived from *miR-21*-transfected normal fibroblasts mimicked the effects of CAF-derived exosomes, in terms of increased capacity of breast cancer cell lines to form mammospheres, augmented stem cell and EMT markers, and enhanced anchorage-independent growth. In addition, these effects were reverted by transfection with anti-miRs (i.e., antisense oligonucleotides complementary to the miRNA of interest), thus confirming the key role of exosomal *miR-21* [63]. In the colorectal cancer context, orthotopic xenografts established with *miR-21*-overexpressing fibroblasts and tumor cells led to increased liver metastases compared to those established with control fibroblasts [64].

With regard to miRNAs found enriched in CAF exosomes, Wang et al. [65], using a miRNA microarray analysis, identified 13 miRNAs that are significantly increased in exosomes derived from osteosarcoma CAFs with respect to the corresponding paracancer fibroblasts, among which *miR-1228*. This miRNA, once transferred to osteosarcoma cells, was shown to promote cell migration and invasion by targeting *SCAI*. Similarly, CAF-derived exosomal *miR-382-5p* was proven to stimulate the migration and invasion of oral squamous cell carcinoma cells [66]. *miR-181d-5p*, vehiculated through exosomes of breast CAFs, was instead reported to enhance the aggressiveness of MCF-7 cells by stimulating proliferation and antagonizing apoptosis, via the suppression of CDX2 transcription factor [67].

The literature also provides plenty of examples of miRNAs that are less enriched in exosomes from CAFs as compared to those from normal fibroblasts, usually tumor-suppressor miRNAs, such as *miR-34a* [68]. This would suggest that in normal conditions, fibroblasts sustain a certain degree of suppression on the growth of epithelial cells, through the exosome-mediated transfer of such miRNAs. Upon fibroblast activation, delivery of tumor-suppressive miRNAs is reduced and the brake imposed on tumor growth released. This class of exosomal miRNAs includes *miR-34a-5p* in oral squamous cell carcinoma [69], *miR-4516* and *miR-1-3p* in breast cancer [70,71], *miR-148b* and *miR-320a* in endometrial cancer [72,73] and *miR-320a* in hepatocellular carcinoma [74]. Notably, for *miR-148b* [72], *miR-1-3p* [71] and *miR-320a* [74], a role in tumor invasion and metastasis was also reported.

Exosomes are not the unique way of horizontal transfer of miRNAs, which instead can circulate within protein/lipoprotein complexes [75] or in other types of vesicles (e.g., oncosomes [76]). For example, Doldi et al. showed that muscle cell-specific *miR-133b* is upregulated in prostate CAFs (either when patient derived or activated in vitro, especially with IL-6) and released by them in the extracellular space predominantly in a non EV-associated form [16]. Soluble *miR-133b* is then efficiently taken up by prostate cancer cells, where it may contribute to establishing a mesenchymal phenotype [35]. Such direct transfer of miRNAs typically expressed in cells of the mesenchymal lineage may represent a further intriguing mechanism by which CAFs can induce EMT in tumor cells.

LncRNAs were also shown to be vehiculated by CAFs to tumor cells through exosomes. This is the case of *H19*, found to be highly expressed in tumor stroma than in cancer nests of colorectal cancer tissues. Interestingly, such lncRNA is highly enriched in CAF-derived exosomes and transferred to colorectal cancer cells, where it promotes stemness and chemoresistance in vitro and in vivo, by increasing the frequency of tumor-initiating cells [77]. Mechanistically, *H19* was shown to act as a molecular sponge for *miR-141*, thus impacting on the β-catenin pathway. CAF-secreted exosomal *SNHG3* lncRNA was instead shown to serve as a sponge for *miR-330-5p* in breast cancer cells. Augmented availability of the miRNA was shown to induce derepression of its target Pyruvate Kinase M1/M2 PKM, with subsequent inhibition of mitochondrial oxidative phosphorylation in favor of glycolysis and the ultimate effect of boosting breast tumor cell proliferation [78].

A comprehensive list of extracellular ncRNAs derived from CAFs and delivered to tumor cells is reported in Table 2 (left).

### 3.2. ncRNAs Released by Tumor Cells to Communicate with CAFs

Although tumor cells can readily release miRNAs in the extracellular space, eventually encapsulated within EVs, and thereby condition surrounding fibroblasts, this field is much less explored as compared to that of CAF-derived miRNAs. Baroni et al. showed that *miR-9*, a known metastamiR (i.e., a pro-metastatic miRNA found upregulated in various breast cancer cell lines), is released by triple negative breast cancer cells in an exosomal form and is able to enhance the transition of normal fibroblasts into CAF-like cells. This happens through *miR-9*-mediated modulation of genes mainly involved in cell motility and extracellular matrix remodeling pathways [89]. Notably, when normal fibroblasts were directly transfected with *miR-9*, they became able to promote the in vivo growth of breast cancer cells [89] and increase cisplatin resistance through EGF containing fibulin extracellular matrix protein 1 (*EFEMP1*) suppression [90].

Proangiogenic properties of CAFs were also shown to be dependent on signals vehiculated by tumor cells through exosomes. Lung cancer-derived exosomal *miR-210* was indeed reported to elevate the expression of proangiogenic factors, such as MMP9, FGF2 and vascular endothelial growth factor (VEGF), in recipient fibroblasts by modulating JAK2/STAT3 signaling pathway and ten-eleven translocation 2 (*TET2*) [86].

Another example of how tumor-derived exosomal miRNAs may corrupt fibroblasts comes from the melanoma dermal niche. Melanocytes were shown to traffic *miR-211* through melanosomes (i.e., melanoma exosomes) to primary dermal fibroblasts, thus enhancing their proliferation, migration and pro-inflammatory gene expression, all known features of CAFs [87]. This would result in the creation of a niche permissive to melanoma invasion through the dermis. Melanosomes were also shown to carry the *Gm26809* lncRNA, which was reported to mediate the reprogramming of NIH/3T3 fibroblasts into CAF-like cells [92], as evidenced by increased expression of activation markers (α-smooth actin and fibroblast activation protein) and cell migration. *POU3F3* lncRNA was also shown to be transferred from esophageal squamous cell carcinoma cells to normal fibroblasts and mediate their activation. In turn, such CAF-like cells were demonstrated to promote proliferation and cisplatin resistance of tumor cells through the secretion of IL-6 [99]. As far as lncRNAs are concerned, direct transfer of tumor-derived *Lnc-CAF* through exosomes was reported to be in part responsible for upregulation of *Lnc-CAF* found in CAFs, as mentioned above [29].

A comprehensive list of extracellular ncRNAs derived from tumor cells and delivered to CAFs is reported in Table 2 (right).

## 4. Targeting ncRNAs to Interrupt Tumor-CAF Interplay

The enormous amount of reports showing the active role of CAFs in supporting tumor progression and ultimately determining the aggressiveness of a cancer cell is per se sufficient, at least from a conceptual point of view, to hypothesize the efficacy of a CAF-directed therapy as an alternative to or in combination with the conventional tumor cell-centered approach [100]. In this regard, ncRNAs shown to play a role in either fibroblast activation or in CAF-induced changes in recipient cancer cells may be regarded as possible therapeutic targets or tools to interrupt tumor-stroma interplay (Figure 1b). This would produce beneficial therapeutic effects in terms of reduced tumor growth and dissemination, which would result from the impairment of processes that are known to be sustained by a proficient interaction with the microenvironment, such as metabolic reprogramming, EMT and angiogenesis (Figure 1a).

Aside from such direct anticancer effects, interruption of tumor-stroma interplay may also increase the response to conventional oncological therapies, in view of the increasingly reported role of CAFs in determining the sensitivity/resistance of tumor cells to a wide spectrum of chemo-, endocrine and radiotherapeutic regimens [101]. For example, CAF exosomes were shown to promote cisplatin resistance of ovarian cancer cells and head and neck cancer cells, through the delivery of *miR-98-5p* and *miR-196a*, respectively [85,96]. Interestingly, the two miRNAs accomplish this effect by inhibiting genes of the same family, namely *CDKN1A* and *CDKN1B*. *CCAL* (colorectal cancer-associated lncRNA), transferred from CAFs to colorectal cancer cells via exosomes, was shown to confer resistance to oxaliplatin through the direct interaction with mRNA stabilizing protein HuR (human antigen R) and the consequent increase of β-catenin [97]. In the context of pancreatic cancer, where acquired resistance to gemcitabine remains a challenge, Fang and colleagues showed that (i) CAFs are innately resistant to gemcitabine, (ii) *miR-106b* is increased in CAFs and derived exosomes upon treatment with the drug, and (iii) *miR-106b* transferred to tumor cells promotes gemcitabine resistance [80]. Interestingly, inhibition of *miR-106b* in CAFs was shown to re-establish tumor cell sensitivity to gemcitabine [80]. In models of luminal breast cancer undergoing treatment with the antiestrogen fulvestrant, *miR-221*, horizontally delivered by CAF microvesicles to tumor cells, was reported to trigger the conversion of noncancer stem cells into estrogen receptor-independent cancer stem cells, thus ultimately promoting hormonal therapy resistance [88]. Chen et al. identified CAF-released exosomal *miR-93-5p* as being responsible for rescuing colorectal cancer cells from radiation-induced apoptosis in vitro and in vivo, suggesting a role in radiotherapy resistance [95]. Similarly, in the esophageal squamous cell carcinoma context, CAFs were shown to contribute to tumor cell radiation resistance through the induction of *DNM3OS* lncRNA, which occurred in a PDGFβ/PDGFRβ/FOXO1 signaling pathway-dependent manner and ultimately impaired DNA damage response [44].

From the therapeutic point of view, an intrinsic advantage of ncRNAs is related to their capability to regulate multiple genes simultaneously, thus becoming valuable targets in the so-called ‘one hit multitarget’ approach. Tools for inhibiting or restoring ncRNA expression for therapeutic purposes have been widely described in both in vitro and in vivo settings (reviewed in [102]), with some of them being tested in clinical trials for oncologic or non-oncologic diseases in humans [103]. Such tools include chemically modified antisense oligonucleotides for the silencing of both miRNAs and lncRNAs or mimics for miRNA overexpression [102] (Figure 1b). It is of note, however, that the two major completed clinical trials with miRNA mimics (ClinicalTrials.gov Identifier: NCT02369198, ClinicalTrials.gov Identifier: NCT01829971) provided contrasting outcomes, especially as regards safety. In addition, overexpression of lncRNAs in vivo remains a challenge [104].

In light of these concerns, our view is that a major progress in the field could be the identification of drug-like small molecules able to target specifically the ncRNAs of interest, as they may guarantee a better pharmacokinetic profile and cost-efficiency as compared to nucleic acid-based oligomers (Figure 1b). In the context of miRNAs, some small molecules have been reported to inhibit miRNA biogenesis by blocking the processing sites for Drosha or Dicer on primary or precursor miRNAs, respectively (reviewed in [105] and in [106]). This is made possible by the fact that secondary motifs, namely hairpin structures, present in such transcripts are available for ligand binding. As for miRNAs reported to play a role in tumor-stroma interplay, precursors of *miR-21* and *miR-210* showed to be efficiently bound by the small molecules mitoxantrone and a bisbenzimide analog called targarpremir-miR-210, respectively, which resulted in impaired processing and reduced mature miRNA availability [105,106]. To our knowledge, no small molecules have been described to target mature miRNAs directly. In this regard, longer RNAs such as lncRNAs are more suited for small molecule targeting. In fact, lncRNAs can locally fold into a variety of secondary structures including helices, hairpins, loops, bulges, G-quadruplexes and pseudoknots, which can then interact to form higher order hierarchical structures [107]. Such structures, besides representing the functional elements of lncRNAs, may also be the scaffolds for the interaction with small molecules, which in turn may favor or impair lncRNA activity [107]. High-throughput screenings or molecular docking experiments showed to be successful approaches for the identification of small molecules able to selectively bind specific RNA moieties present in precursor/primary miRNAs or lncRNAs (reviewed in [107] and in [108]). For example, through high-throughput molecular docking-based virtual screening of the PubChem library, the small compounds AC1Q3QWB and AC1NOD4Q were revealed to specifically interfere with *HOTAIR* function by inhibiting the interaction with EZH2 [109,110].

Waiting for the advent of specific ncRNA-targeting small molecules, research in the field of miRNAs or lncRNAs may also allow to identify pathways relevant for both fibroblast activation and for the subsequent tumorigenic spur induced on tumor cells. It is likely that drugs targeting most such pathways may already exist, even if used for other purposes. These molecules could be tested for their ability to interrupt tumor-stroma interplay and quite easily moved to the clinic in the context of drug repurposing or repositioning approaches.

## 5. Tumor-CAF Interplay-Related ncRNAs as Biomarkers

**Figure 2:**
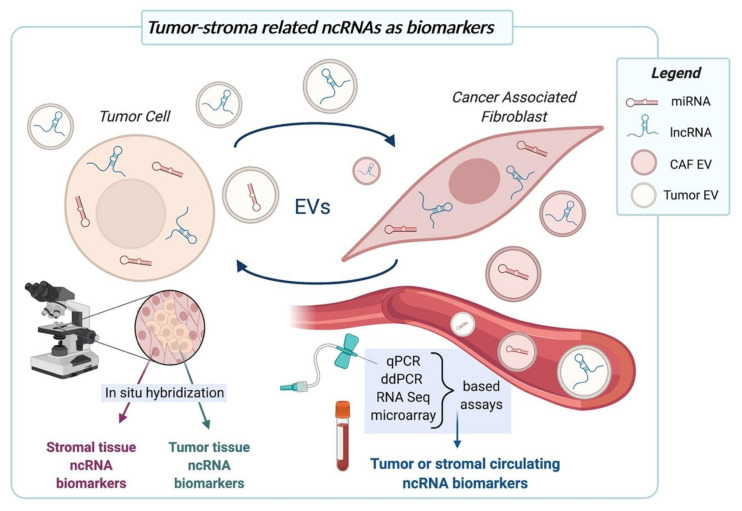
ncRNAs shown to play a role in tumor-stroma interplay may be regarded as possible prognostic or predictive biomarkers. Tissue ncRNAs can be separately assessed by ISH in cancer cells and CAFs in biopsies or surgical specimens. Extracellular ncRNAs exchanged between tumor cells and CAFs can leak into the circulation and be measured by microarray-, PCR- or sequencing-based approaches in liquid biopsies. Created with BioRender.com.

The direct role played by CAFs in determining tumor cell aggressiveness and in particular the fact that acquisition of the metastatic competence is intimately associated with the establishment of a proficient tumor-stroma interplay suggest that prognostic information may be derived not only from tumor cell-related but also from CAF-related factors. For instance, the expression levels of ncRNAs found to be modulated in either type of cell upon mutual interaction may be used to predict tumor behavior and patient outcome (Figure 2). In this regard, several reports highlight that stromal *miR-21* expression has even higher prognostic significance than that from tumor cells. Proof of this is a study conducted on gastric cancer tissue sections from about 500 patients, where the expression of *miR-21* was assessed separately in tumor cells and stromal cells using in situ hybridization (ISH) [23]. Through this analysis, the authors found that, though *miR-21* was highly expressed in both compartments, *miR-21* of tumor cells was not related to clinicopathological factors, whereas stromal *miR-21* was associated with tumor stage, size, and nodal metastasis. In addition, stromal *miR-21* gradually increased during tumor progression. These findings were confirmed by Kunita et al. on a series of 89 invasive lung adenocarcinoma cases, where higher *miR-21* levels in CAFs, but not in cancer cells, were associated with a reduced patient survival [22]. A prognostic significance was also reported for CAF-related lncRNAs. In this regard, high stromal *TIRY* expression in a prospective cohort of oral squamous cell carcinoma specimens was associated with tumor stage, metastasis status and poor prognosis, in terms of a shorter progression-free survival of patients [28]. In the same tumor context, high *Lnc-CAF* expression in CAFs correlated with high TNM stage and predicted poor prognosis [29].

From a technical point of view, the methodology that can be more easily implemented in the histopathology routine to measure stromal ncRNAs is ISH (Figure 2). Labelled complementary probes may hence bind to the ncRNA of interest in both frozen and formalin-fixed paraffin embedded tissue sections, and the signal from stromal and tumor cells can be analyzed independently to assess the specific prognostic value. In contrast, PCR-based techniques on bulk tissues would not distinguish ncRNA signal coming from the different cell components.

ISH can also be used on sections from both surgical specimens and biopsies. The fact that tumor-stroma interplay does not only rely on direct cell-cell contact but also on paracrine signals and EVs allows to predict that ncRNA changes may be detected in non-neoplastic areas adjacent to the frank tumor mass. In light of this, we may speculate that in the context of random systematic bioptic procedures, tumor-negative biopsies may also carry relevant prognostic information or even allow the diagnosis of a still occult (i.e., not biopsied) cancer.

The evidence collected on the role of tumor-stroma interplay in therapy resistance highlights that stromal ncRNAs may also be used as predictive biomarkers to determine the sensitivity of a given tumor to specific therapies upfront or to monitor tumor response/acquisition of resistance during treatment. For instance, Zhang et al. found that pancreatic ductal adenocarcinoma patients who were resistant to gemcitabine tended to have higher *miR-21* expression and more activated CAFs in tumor stroma [21].

As outlined in the review, CAFs and tumor cells often communicate through the horizontal transfer of EVs, which were shown to contain both miRNAs and lncRNAs. Such vesicles deliver ncRNAs not only locally but, once released into the bloodstream, can also influence tumor progression at distant sites (Figure 2). From the biomarker point of view, the possibility to catch tumor-stroma related signals in circulation has tremendous impacts. First of all, liquid biopsy is the best source of biomarkers for its non-invasive nature and applicability to monitoring scenarios, such as tracking of tumor progression or treatment response. Liquid biopsies also make it possible to get information from different tumor foci simultaneously (even from occult tumors) and on the host systemic response to cancer. In this context, high levels of plasma exosomal *miR-196a* were shown to be correlated with poor overall survival and chemoresistance in head and neck cancer patients [85]. Similarly, microvesicles from patients with hormone-treatment resistant metastatic breast cancer expressed high levels of *miR-221* [88], thus making this miRNA a possible predictive or monitoring biomarker. High levels of plasma exosomal lncRNA *POU3F3* correlated significantly with lack of complete response to cisplatin and poor survival in esophageal squamous cell carcinoma patients [94].

From a methodological point of view, circulating ncRNAs can be easily quantified using a variety of methods, including quantitative PCR (qPCR), digital droplet PCR (ddPCR), microarray-based approaches and RNA sequencing [111,112,113] (Figure 2). Favorable to the measurement of ncRNAs in body fluids is the fact that EVs protect ncRNAs from degradation, thus increasing their stability. Obviously, clinical-grade biomarker discovery needs efforts in planning well designed studies in terms of patient cohorts, assessment of clinical outcomes and methodology. In particular, standardized procedures should be set up from sample choice (plasma vs. serum, EVs vs. non-EV enriched fraction) and collection, to ncRNA quantification (methods and platforms) and subsequent management and normalization of data [111,112,113].

## 6. Conclusions

The literature provides plenty of evidence regarding the tremendous impact of miRNAs in regulating different aspects of tumor-stroma interplay, with obvious therapeutic implications. In this scenario, we may expect that new discoveries on lncRNAs may unearth additional clinically relevant links between ncRNA function in CAFs and tumor cells. From the therapeutic point of view, the armamentarium for the direct targeting of ncRNAs, which ranges from nucleic acids to small molecule-based approaches, is continuously expanding (Figure 1b). Research on ncRNAs may also reveal novel actionable pathways that can be targeted to interrupt tumor-stroma crosstalk using already existing molecules in drug repositioning or repurposing approaches. From the biomarker side, the growing body of evidence regarding the prognostic/predictive relevance of stromal ncRNAs, both in tissues and in circulating exosomes, suggests their possible application as tools to assist histopathology routine or as entities to be measured in liquid biopsies, respectively (Figure 2).

Study of tumor-stroma interplay has also made it possible to raise concerns with respect to well-established concepts, such as the oncogenic/tumor-suppressive role or the biomarker value of ncRNAs found to be deregulated in human tumors. For example, although *miR-21* has been shown to be upregulated in almost all types of cancer and reported to have a genuine oncogenic function when ectopically induced [114], several studies are now showing that stromal *miR-21* has a far higher prognostic value than the tumoral one. This may suggest that *miR-21* overexpression found in human cancers is at least in part the result of an increased expression of the miRNA in the stroma, for example in CAFs; in addition, the oncogenic role of *miR-21* may rely on its stromal rather than epithelial function. In line with this, at least in some tumor types, specific inhibition of *miR-21* in tumor cells was shown to be devoid of therapeutic benefits [115]. Altogether, these findings should increase appreciation of the pivotal role of tumor microenvironment and help to change the paradigm from a tumor cell-centric to a more tumor/stroma-oriented view of cancer biology, with obvious implications for both biomarker and therapy development.

## Figures and Tables

**Table 1:** Intracellular ncRNAs reported to have a role in tumor-stroma interplay.

Intracellular ncRNAs in CAFs			Intracellular ncRNAs in Tumor Cells		
ncRNA	Tumor Type	Reference	ncRNA	Tumor Type	Reference
*let-7b*	breast cancer	[30]	*miR-101*	hepatocellular carcinoma	[31]
*miR-101*	hepatocellular carcinoma	[31]	*miR-1247*	prostate cancer	[32]
*miR-133b*	prostate cancer	[16]	*miR-200b*	gastric cancer	[33]
*miR-145*	oral cancer	[19]	*miR-205*	prostate cancer	[34]
*miR-155*	ovarian cancer	[15]	*miR-29b*	ovarian cancer	[35]
*miR-200 family*	ovarian cancer; breast cancer	[36,37,38]	*miR-33b*	lung cancer	[39]
*miR-205*	breast cancer	[40]	*ANRIL*	oral squamous cell carcinoma	[41]
*miR-21*	pancreatic adenocarcinoma; lung adenocarcinoma; gastric cancer; breast cancer; colorectal cancer	[20,21,22,23,24,42,43]	*DNM3OS*	esophageal squamous cell carcinoma	[44]
*miR-210*	prostate cancer	[18]	*HOTAIR*	breast cancer	[45]
*miR-214*	ovarian cancer	[15]	*LINC00092*	ovarian cancer	[46]
*miR-221*	breast cancer	[37]	*UCA1*	colorectal cancer; glioblastoma	[47,48]
*miR-222*	breast cancer	[49]	*ZEB2NAT*	bladder cancer	[50]
*miR-27*	esophageal cancer	[51]			
*miR-31*	colorectal cancer; ovarian cancer; endometrial cancer	[15,52,53]			
*TIRY*	oral squamous cell carcinoma	[28]			
*lnc-CAF/FLJ22447*	oral squamous cell carcinoma	[29]			

**Table 2:** Extracellular ncRNAs reported to have a role in tumor-stroma interplay.

Extracellular ncRNAs from CAFs			Extracellular ncRNAs from Tumor Cells		
ncRNA	Tumor Type	Reference	ncRNA	Tumor Type	Reference
*miR-1*	breast cancer	[71]	*miR-105*	breast cancer	[79]
*miR-106b*	pancreatic cancer	[80]	*miR-1247*	hepatocellular carcinoma	[81]
*miR-1228*	osteosarcoma	[65]	*miR-125b*	triple negative breast cancer	[82]
*miR-148b*	endometrial cancer	[72]	*miR-142-3p*	lung cancer	[83]
*miR-181d-5p*	breast cancer	[67]	*miR-155*	pancreatic adenocarcinoma	[84]
*miR-196a*	head and neck cancer	[85]	*miR-210*	lung cancer	[86]
*miR-21*	breast cancer; colorectal cancer	[63,64]	*miR-211*	melanoma	[87]
*miR-221*	endocrine resistant breast cancer	[88]	*miR-9*	breast cancer	[89,90]
*miR-3188*	head and neck cancer	[91]	*Gm26809*	melanoma	[92]
*miR-320a*	endometrial cancer; hepatocellular carcinoma	[73,74]	*PCAT-1*	lung cancer	[93]
*miR-34a-5p*	oral squamous cell carcinoma	[69]	*POU3F3*	esophageal cancer	[94]
*miR-382-5p*	oral squamous cell carcinoma; colorectal cancer	[64,66]			
*miR-4516*	triple negative breast cancer	[70]			
*miR-93-5p*	colorectal cancer	[95]			
*miR-98-5p*	ovarian cancer	[96]			
*CCAL*	colorectal cancer	[97]			
*H19*	colorectal cancer	[77]			
*SNHG3*	breast cancer	[78]			
*UCA1*	vulvar squamous cell carcinoma	[98]			
